# Effects of bacitracin methylene disalicylate and diet change on gastrointestinal integrity and endotoxin permeability in the duodenum of broiler chicken

**DOI:** 10.1186/s13104-017-2781-8

**Published:** 2017-09-08

**Authors:** Dawn A. Koltes, Howard D. Lester, Maurice Frost, Douglas Aldridge, Karen D. Christensen, Colin G. Scanes

**Affiliations:** 1 Department of Poultry Science, University of Arkansas, 1260 W. Maple, POSC O-215, Fayetteville, AR 72701 USA; 2 Hy-line International, Des Moines, IA 50309 USA

**Keywords:** Duodenum, Bacitracin methylene disalicylate, Grower diet, Starter diet

## Abstract

**Objective:**

To determine the effect of bacitracin methylene disalicylate (BMD) and feed changes on gastrointestinal integrity, endotoxin permeability, and morphometric parameters in the duodenum of broilers.

**Results:**

Birds were raised on a starter diet without growth promoting antibiotics for 31 days then switched to a grower diet. Four of the pens including 50 g/ton of BMD while 4 pens remained antibiotic free. Eight birds per treatment were sampled prior to the feed change and at 3 and 7 days following the feed change. Gastrointestinal integrity and endotoxin permeability in the duodenum were determined using a modified Ussing Chamber and an adjacent section fixed in 10% formalin for morphometric analysis. Data were analyzed using Proc Glimmix of SAS with the model fitting BMD treatment, time, and the interaction of BMD treatment and time as fixed effects. Intestinal integrity increased at d 3 and 7 compared to prior to the feed change and addition of BMD (P > 0.001) and villus height was decreased with BMD supplementation (*P* = 0.049). All other tested effects similar (*P* > 0.1). In conclusion, the practice of changing feed had a greater effect on intestinal health than addition of BMD. However, the factors driving these differences 42 are unclear.

**Electronic supplementary material:**

The online version of this article (doi:10.1186/s13104-017-2781-8) contains supplementary material, which is available to authorized users.

## Introduction

Antibiotics have been used in agricultural diets to improve animal performance for several decades [1]. However, concern over antibiotic resistant pathogens has increased the need to identify safe non-antibiotic alternatives [1]. While alternative products have been identified that promote similar growth performance, these products, as a whole, are often not consistent [2–5]. Despite several decades of use, the mode of action for increase growth performance from the use of sub-therapeutic antibiotics is not fully understood. It has been established that the effects of increased performance are limited to modulation of the microfloral communities and/or the interaction of the microfloral communities with the host with animals raised under sterile conditions do not having elevated performance when given oral antibiotics [6, 7], and the removal from hygienic environments can improve the efficacy of sub-therapeutic antibiotics.

Sub-therapeutic antibiotics are known to alter gastrointestinal morphology, weight, length, and integrity [6, 8–11]. However, supplementation starts at hatch and it is unclear the effects of supplementation later in life. Development of the gastrointestinal tract and microbial comminutes within the gastrointestinal tract are generally stabilized at approximately 3 weeks of age [12–14]. Therefore, we wanted to determine if the use of a commonly used broad-spectrum antibiotic, bacitracin methylene disalicylate (BMD) in birds raised without antibiotics would alter gastrointestinal integrity, endotoxin permeability, or morphometric analysis in a stable gastrointestinal tract.

## Main text

### Methods

#### Animals

On day of hatch, broilers were vent sexed and 22 males were randomly placed in each of 8 1.22 m by 1.22 m floor pens at the Arkansas Division of Agriculture Applied Broiler Research Unit (Savoy, Arkansas). Broilers were checked twice daily for unhealthy birds and sufficient feed in tube feeders. For the first 8 days, broilers were raised under continuous lighting and then switched to a schedule of 16 h of light: 8 h of dark until processing. Broilers were allowed free access to feed and water throughout the study. Broilers were maintained on an antibiotic free diet for 31 days at which point 4 of the floor pens received a diet containing 50 g/ton of bacitracin methylene disalicylate (BMD). Diet formulas are provided in Table 1.

**Table 1:** Components of the starter diet and grower diet with and without bacitracin methylene disalicylate

Ingredients (%)	Starter^b^	Grower^c^	
	Without BMD	Without BMD	With BMD
Corn	63.07	66.43	66.43
Soybean meal	25.75	26.80	26.80
Fat	2.85	1.13	1.13
Calcium carbonate	1.03	0.33	0.33
Deflourinated phosphate	0	1.36	1.36
Dicalcium phosphate	1.10	0	0
Sodium chloride	0.40	0.37	0.37
DL methionine (99.5%)	0.28	0.22	0.22
Trace minerals	0.10	0.10	0.10
Choline chloride (60%)	0.22	0.00	0.00
Vitamin premix	0.20	0.50	0.50
ProPack^a^	5.00	0.00	0.00
Pro-Plus^a^ + Thr and Lys	0.00	2.76	2.76
BMD	0.00	0.00	0.0055

^a^Protein concentrates from H.J. Baker

^b^The starter basal diet was fed from day of hatch through 30d post hatch

^c^The grower basal diet was fed beginning on day 31 after baseline (day 0) birds were samples

#### Tissue collection

Prior to the addition of BMD (0 days) and on days 3/4 and 7/8 of BMD inclusion in the diet, individual body weights were collected on 8 (2 randomly chosen birds per pen) birds per treatment. The sample size was selected based on a power analysis and the number of Ussing chambers available. Sampling was split across 2 days with equal numbers of birds from each treatment sampled on each day. Birds were euthanized using cervical dislocation, and a 5 cm section of the descending duodenum was collected, flushed with Krebs-Heinchlet buffer, [11.1 mM dextrose, 1.2 mM Magnesium sulfate, 1.2 mM Potassium phosphate monobasic, 4.7 mM Potassium chloride, 118 mM Sodium chloride, 1 mM Calcium chloride dehydrate, 25 mM Sodium Bicarbonate; pH 7.4], stored in ice cold Krebs-Heinchlet buffer under atmospheric aeration, and transported back to the laboratory to undergo integrity and permeability assays using an easy mount Ussing chamber. A small adjacent section was collected and stored in formalin until histological analysis.

#### Gastrointestinal integrity and endotoxin permeability

Gastrointestinal integrity as measured by transepithelial electrical resistance (TEER) and endotoxin permeability using previously published methods [10]. Briefly, the serosal layer of the duodenum was removed, the intestine was opened and mounted on an 0.3 cm^2^ easy mount Ussing chamber (Physiological Instruments, San Diego, CA). The apical and basolateral sides of the tissue were immersed in Krebs-Heinchlet buffer and samples were kept at 39 °C and under continuously aeration with 5% carbon dioxide. Basal resistance was recorded for 10–15 min using the Acquire and Analyze software.

Immediately following collection of basal TEER, buffer was removed and 20 µg/ml of fluorescein isothiocyanate labelled lipopolysaccharide (FITC-LPS) from *Escherichia coli* 0111:B4 (Sigma Aldrich, F3665, St. Louis, MO) was applied to the apical side. Basolateral FITC-LPS concentrations were determined every 15 min, using a Synergy HTX fluorescence spectrophotometer (BioTek US, Winooski, VT) with an excitation and emission wavelengths of 485 and 528 nm, respectively. An apparent permeability co-efficient (Papp) was calculated for each tissue as follows:

$${\text{Papp}} = {\text{dQ}}/ ({\text{dt}} \times {\text{A}} \times {\text{C}}_{0} ).$$

where dQ/dt is the transport rate (µg/s) and corresponds to the slope of the regression line, C_0_ is the initial concentration in the mucosal side of the chamber (mg/ml) and A is the area of the membrane (0.3 cm^2^) [10, 15].

#### Histology

Formalin fixed duodenal samples underwent paraffin embedding, sectioning and staining with hematoxylin and eosin at the University of Arkansas microscopy laboratory [16]. Five images per slide of were captured using a Zeiss Imager M2 microscope (Carl zeiss microscopy, LLC., Thornwood, NY) with attached CCD camera (Hamamatsu, Orca ER, Bridgewater, NJ) and Image-Pro Plus software (Media cybernetics, Inc., Rockville, MD). Villus height, crypt depth and muscularis thickness (n = 5 per bird) were measuring using Image J software [17, 18].

#### Statistical analysis

All data were analyzed using mixed models through the Proc Glimmix package of the SAS software [19] with BMD treatment, day of treatment, and the interaction of BMD treatment and day of treatment fit as fixed effects. For models accounting for weight, the Proc Glimmix package of the SAS software [19] with BMD treatment, day of treatment, and the interaction of BMD treatment and day of treatment fit as fixed effects, and individual body weight was included as a covariate. Residuals were tested for normality and were found to be normally distributed. Least square means were determined using the LSMeans statement of SAS. If the main treatment effect was significant (*P* < 0.05), pairwise comparisons were estimated using the pdiff statement in SAS followed by Tukey post hoc adjustment to account for multiple tests in the model [19].

### Results

#### Growth performance

As expected in growing male broiler chickens, body weight linearly increased over time (*P* < 0.001). Body weight increased from day 0 to day 3 (*P* < 0.001) and then again from day 3 to day 7 (*P* < 0.001; Table 2). Neither BMD supplementation (*P* = 0.233) nor the interaction between day and BMD supplementation (*P* = 0.583) altered body weight.

**Table 2:** Changes in body weight, gastrointestinal integrity, and endotoxin permeability over time

	Unit	N	LSMean	SEM	*P* value
Body weight					<0.001
Day 0^a,b^	Kg	8	1.59^x^	*0.04*	
Day 3	Kg	16	2.10^y^	*0.04*	
Day 7	Kg	16	2.53^z^	*0.04*	
Gastrointestinal integrity					<0.001
Day 0	Ω/cm^2^	8	396.3^z^	*14.3*	
Day 3	Ω/cm^2^	14^c^	537.5^y^	*14.1*	
Day 7	Ω/cm^2^	14^c^	519.4^y^	*13.6*	
Endotoxin permeability					0.903
Day 0	µg/ml/min/cm^2^	8	3395	*2016*	
Day 3	µg/ml/min/cm^2^	16	5066	*1886*	
Day 7	µg/ml/min/cm^2^	16	5808	*1615*	

^a^ Day 0 (31 days post hatch) samples were taken as baseline samples prior to the switch in feed to a grower diet on day 0 that either contained 50 g/ton of bacitracin methylene disalicylate (BMD) or no BMD. Day 3 samples were taken 3 and 4 days following the feed change and occurred at 34 and 35 days post hatch, respectively. Day 7 samples were taken 7 and 8 days following the feed change and occurred at 38 and 39 days post hatch, respectively

^b^ Data were similar for BMD dietary treatment and the interaction of day and BMD treatment; therefore, data shown are for differences in day regardless of dietary treatment

^c^ Due to malfunctions with the chambers data was only collected on 7 broilers per treatment with a total of 14 measurements collected for each time point

^xyz^ changes in superscript within row indicate differences between pairwise comparisons after Tukey’s post hoc adjustment (*P* < 0.05)

#### Gastrointestinal measurements

Gastrointestinal integrity of the duodenum as measured by transelectrical epithelial resistance (TEER) was different over the course of the study (*P* < 0.001) with TEER increasing at day 3 and day 7 compared to day 0 (*P* < 0.001; *P* < 0.001, respectively; Table 2). Gastrointestinal integrity was not altered by supplementation with BMD (*P* = 0.938) or the interaction between day and BMD supplementation (*P* = 0.876). Endotoxin permeability was not different with day (*P* = 0.903), BMD supplementation (*P* = 0.340), or the interaction between day and BMD supplementation (*P* = 0.457).

Morphometric analysis was conducted on adjacent segments of the duodenum used for gastrointestinal integrity and endotoxin permeability assays. Villus height decreased in birds supplemented BMD (*P* = 0.050), but was not different between day (*P* = 0.163), or the interaction between day and BMD supplementation (*P* = 0.128; Table 3). Crypt depth, villus height to crypt depth ratio, and muscularis depth were not different across day, BMD supplementation, and the interaction between day and BMD supplementation (*P* > 0.05). Total thickness (villus height + crypt depth + muscularis depth) increased at day 7 compared to day 3 (2719.88 ± 70.185 and 2431.58 ± 70.185, respectively; *P* = 0.007), but was similar between BMD supplementation (*P* = 0.130) and the interaction between dietary BMD supplementation and day (*P* = 0.311). All *P* values are included in Additional file 1: Table S1.

**Table 3:** Changes in duodenal morphometric parameters following short term exposure to bacitracin methylene disalicylate

	Unit	N	LSMean	SEM	*P* value
Villus height					0.049
BMD^a^	µm	16	2018.6^x^	*49.5*	
ABF	µm	16	2162.8^y^	*49.5*	
Crypt depth					0.746
BMD	µm	16	384.8	*15.3*	
ABF	µm	16	391.9	*15.3*	
Ratio					0.339
BMD	µm/µm	16	5.6	*0.2*	
ABF	µm/µm	16	6.0	*0.2*	
Muscularis thickness					0.348
BMD	µm	16	193.6	*8.7*	
ABF	µm	16	205.3	*8.7*	
Total thickness					0.130
BMD	µm	16	2498.4	*70.2*	
ABF	µm	16	2653.1	*70.2*	

*BMD* broilers that received feed with 50 g/ton of bacitracin methylene disalicylate beginning at 31 days post hatch, *ABF* treatment group where broilers received no bacitracin methylene disalicylate; Ratio, villus height to crypt depth ratio; Total thickness, villus height + crypt depth + muscularis depth

^a^Data were similar for day and the interaction of day and BMD treatment; therefore, data shown are for differences in BMD dietary treatment where data from day 3 and 7 are combined

^xyz^changes in superscript within row indicate differences between pairwise comparisons after Tukey’s post hoc adjustment (*P* < 0.05)

Given body weight was significantly different over the course of the study, and can influence gastrointestinal measurements, we chose to run additional analysis to determine if differences in body weight contribute to the overall changes in TEER, endotoxin permeability, or morphometric analysis. When body weight is included as a covariate, endotoxin permeability was different across day (*P* = 0.006), increasing at day 7 compared to day 0 (*P* = 0.019). Neither treatment with BMD (*P* = 0.338), nor interaction of BMD supplementation and day (*P* = 0.440) were significant. Addition of body weight to the statistical model, did not alter results observed previously for TEER, but did removed differences in BMD treatment or day effects observed for morphometric analysis. See Additional file 1: Table S2 for *P* values.

### Discussion

With the use of antibiotics being phased out of animal agriculture, alternatives need to be identified to allow for healthy, efficient animal agriculture. Many alternative have been identified, such as prebiotics and probiotics, however; success rates vary greatly. Therefore, identifying the underlying modes of action for sub-therapeutic antibiotics will be important for the development of alternatives. This study was conducted to understand the effects of sub-therapeutic antibiotics on a stable gastrointestinal tract/microbial community during a mild and routine stressor in poultry production. From our study, only villus height was altered by the use of sub-therapeutic antibiotics, and neither gastrointestinal integrity nor endotoxin permeability are consistently effected by sub-therapeutic antibiotics, but differences were observed when the feed was changed from the starter to grower diet.

Decreased villus height along with increased crypt depth is generally indicative poor intestinal function and has associated with exposure to toxin [20]. In our study, we observed a decrease in villus height with the addition of sub-therapeutic antibiotics, but no change in crypt depth or the ration of villus height to crypt depth. This is consistent with results observed by Miles et al. [9]. This slight decrease in villus height initially with supplementation with sub-therapeutic antibiotics may be due to evolving microbial communities during the initial exposure to sub-therapeutic antibiotics. Growth promoting antibiotics have been shown to decrease Gram positive bacteria in poultry [21]. As a result, the proportion of gram negative bacteria increase and allow for increased exposure to lipopolysaccharide, a component of the gram-negative cell wall which can be used to mimic endotoxic stress [21]. However, in our study, we did not observe differences in other measures of enteric health (e.g. TEER, endotoxin permeability), and the effect of BMD treatment on villus height was lost when body weight was added into the statistical model. Therefore, the change we observed in villus height may reflect differences in individual growth rates of the birds given the small sample size.

Duodenal integrity (TEER) and endotoxin permeability were not consistently altered with BMD supplementation and confirm limited changes to the host in response to the addition of sub-therapeutic antibiotics added to the diet late in production. This is the first study to explore the effects of integrity or endotoxin permeability poultry to our knowledge. In swine, whole gastrointestinal permeability decreased with the use of antibiotics based on urinary lactulose to mannitol ratios [22]. While we did not measure whole gastrointestinal permeability, we expected some changes in duodenal integrity or endotoxin permeability. However, the section of the gastrointestinal tract that we focused is not known for high nutrient rates of absorption [23] and may reflect differences in looking at the whole gastrointestinal tract versus a single location and differences between species.

A change was noticed in TEER and endotoxin permeability when individual body weight was included in the statistical model with the change from starter to grower diet. Major differences between these diets included a reduction in DL methionine, fat and calcium carbonate, a complete removal of choline chloride, and an increase in vitamins. While several of these nutrients have been associated with changes in intestinal integrity and permeability in poultry [24], humans [25], swine [26], and rodents [27, 28]. Increasing fat inclusion rates has been a primarily linked to decreased intestinal integrity and increased permeability in mice [27, 28] and poultry [24]. While fat inclusion rates were not as high as in previous studies, gastrointestinal integrity increased and endotoxin permeability decreased with the decreased fat content suggesting this could be a contributing factor to gastrointestinal health in poultry.

## Limitations

While this study identified potential underlying mechanisms that alter poultry gastrointestinal health, the lack of performance data, and age matched samples on the different production diets (grower versus starter; and antibiotic free from hatch to BMD from hatch) limits interpretations and thus warrants additional studies to fully understand mechanisms that contribute to gastrointestinal health in poultry.

## Additional file

**Additional file 1: Table S1.** *P* values for the inclusion of antibiotics in the diet for morphometric changes in duodenum. **Table S2.** *P* values for duodenal traits with body weight included in the model.
