# Supplementary material for: Effects of bacitracin methylene disalicylate and diet change on gastrointestinal integrity and endotoxin permeability in the duodenum of broiler chicken
Source: BMC Res Notes. 2017 Sep 8;10:470. doi: 10.1186/s13104-017-2781-8 (PMC5591554; doi:10.1186/s13104-017-2781-8)
Supplement: Supplementary file 1 — Additional file 1: Table S1. P values for the inclusion of antibiotics in the diet for morphometric changes in duodenum. Table S2. P values for duodenal traits with body weight included in the model. [file 13104_2017_2781_MOESM1_ESM.docx]

**Table S1.** *P-*values for the inclusion of antibiotics in the diet for morphometric changes in duodenum.

|  | **Day**^a^ | | **BMD**^b^ | | **Day*BMD** |
| --- | --- | --- | --- | --- | --- |
| **Villus height** | | 0.163 | | 0.049 | 0.128 |
| **Crypt depth** | | 0.910 | | 0.746 | 0.544 |
| **Ratio** | | 0.835 | | 0.339 | 0.376 |
| **Muscularis depth** | | 0.204 | | 0.348 | 0.500 |
| **Total thickness** | | 0.007 | | 0.130 | 0.311 |

^a^Day 0 samples were taken as baseline samples prior to the feed change from starter to grower diet and either the inclusion of bacitracin methylene disalicylate (BMD) at 50 g/ton or no BMD at 31 days post hatch. Day 3 samples were taken 3 and 4 days following the feed change and occurred at 34 and 35 days post hatch, respectively. Day 7 samples were taken 7 and 8 days following the feed change and occurred at 38 and 39 days post hatch, respectively.

^b^Abbreviations: BMD, identification of treatment effect of 50g/ton bacitracin methylene disalicylate; Ratio, villus height to crypt depth ratio; Total thickness, villus height + crypt depth + muscularis depth

**Table S2.** *P-*values for duodenal traits with body weight included in the model.

|  | **Day**^a^ | | **BMD**^b^ | | **Day*BMD** | **Body Weight** | |
| --- | --- | --- | --- | --- | --- | --- | --- |
| **TEER** | | <0.001 | | 0.876 | 0.791 | 0.206 |  |
| **Papp** | | 0.006 | | 0.338 | 0.441 | <0.001 |  |
| **Villus height** | | 0.318 | | 0.063 | 0.131 | 0.823 |  |
| **Crypt depth** | | 0.184 | | 0.985 | 0.446 | 0.120 |  |
| **Ratio** | | 0.787 | | 0.309 | 0.409 | 0.627 |  |
| **Muscularis depth** | | 0.340 | | 0.340 | 0.464 | 0.792 |  |
| **Total thickness** | | 0.055 | | 0.185 | 0.352 | 0.769 |  |

^a^Day 0 samples were taken as baseline samples prior to the feed change from starter to grower diet and either the inclusion of bacitracin methylene disalicylate (BMD) at 50 g/ton or no BMD at 31 days post hatch. Day 3 samples were taken 3 and 4 days following the feed change and occurred at 34 and 35 days post hatch, respectively. Day 7 samples were taken 7 and 8 days following the feed change and occurred at 38 and 39 days post hatch, respectively.

^b^Abbreviations: BMD, identification of treatment effect of 50g/ton bacitracin methylene disalicylate; TEER, transepithelial electrical resistance as a measure for duodenum integrity; Papp, measure of endotoxin permeability; Ratio, villus height to crypt depth ratio; Total thickness, villus height + crypt depth + muscularis depth
